# Nuclear Export in Non-Hodgkin Lymphoma and Implications for Targeted XPO1 Inhibitors

**DOI:** 10.3390/biom13010111

**Published:** 2023-01-05

**Authors:** Kyla L. Trkulja, Farheen Manji, John Kuruvilla, Rob C. Laister

**Affiliations:** 1 Institute of Medical Science, University of Toronto, 27 King’s College Circle, Toronto, ON M5S 1A1, Canada; 2 Division of Medical Oncology and Hematology, Princess Margaret Cancer Centre, University Health Network, 610 University Avenue, Toronto, ON M5G 2C1, Canada

**Keywords:** lymphoma, hematology, cancer, nuclear export, targeted therapy, proteomics

## Abstract

Exportin-1 (XPO1) is a key player in the nuclear export pathway and is overexpressed in almost all cancers. This is especially relevant for non-Hodgkin lymphoma (NHL), where high XPO1 expression is associated with poor prognosis due to its oncogenic role in exporting proteins and RNA that are involved in cancer progression and treatment resistance. Here, we discuss the proteins and RNA transcripts that have been identified as XPO1 cargo in NHL lymphoma including tumour suppressors, immune modulators, and transcription factors, and their implications for oncogenesis. We then highlight the research to date on XPO1 inhibitors such as selinexor and other selective inhibitors of nuclear export (SINEs), which are used to treat some cases of non-Hodgkin lymphoma. In vitro, in vivo, and clinical studies investigating the anti-cancer effects of SINEs from bench to bedside, both as a single agent and in combination, are also reported. Finally, we discuss the limitations of the current research landscape and future directions to better understand and improve the clinical utility of SINE compounds in NHL.

## 1. Introduction

Mammalian cells are segregated and organized into different compartments in part through the use of lipid membranes. The deployment of membranes in this manner creates functional hubs that separates processes such as energy production (mitochondria), from proteins/fat synthesis and calcium storage (endoplasmic reticulum) and containment of genetic material (nucleus). The correct spatiotemporal localization of molecules is controlled by a variety of mechanisms and a failure to compartmentalize molecules in time and space can lead to diseased states. The nuclear compartment is separated from the cytosol by the nuclear envelope and transport between these two locales is regulated by the nuclear pore complex (NPC). The NPC is a semi-selective barrier that allows the free passage of molecules between 40 and 60 kDa and requires active transport mechanisms for molecules exceeding this molecular weight limit [1]. Given the distinct roles of these compartments, managing transport between the cytoplasm and the nucleus is critical for maintaining cellular homeostasis. The NPC ensures that elements essential for maintaining genomic fidelity remain in the nucleus as required. In the case of possible underlying mechanisms of lymphomagenesis, it is essential that the DNA repair machinery be localized in the nuclear compartment to detect and repair genetic lesions that could otherwise contribute to the acquisition of potentially oncogenic mutations and genetic instability in general. In this context, the aberrant mislocalization of nuclear tumour suppressor proteins, such as p53, have been shown to be potential mechanisms leading to the development of cancers such as lymphoma. The aberrant export of p53 out of the nucleus negates its ability both to effectuate DNA checkpoints/repair and to evoke transcriptional programs triggering cell cycle arrest or apoptosis when needed [2]. Should this occur, potentially oncogenic mutations can be carried forward into daughter cells during cell division/DNA replication. The case of p53 is but one of many examples by which nuclear export can be compromised or coopted to drive processes that have the potential to lead to oncogenesis or sustain tumour growth. In recognition of the fundamental importance of nuclear–cytoplasmic shuttling, much work has been done to identify and characterize the biology of the key players involved in regulating the transfer of biomacromolecules into and out of the nucleus. The knowledge garnered from these studies has laid a foundation for drug development programs to generate targeted agents to inhibit nuclear export as a strategy to treat non-Hodgkin lymphoma (NHL) and other hematological malignancies.

The molecules requiring active transport into and out of the nucleus rely on the activity of the karyopherins which constitutes a family of proteins that handle both nuclear import and export functions [3]. Exportin-1 (XPO1; also known as CRM1) is one of the most comprehensively studied of the nineteen known karyopherin-β family members. The XPO1 cycle modulates the translocation of molecules from the nucleus to the cytoplasm and back in an energy dependent process tightly regulated by the GTP bound form of the Ras-related nuclear protein GTP (RanGTP) gradient which is approximately two orders of magnitude higher in the nucleus as compared to the cytoplasm. This gradient is preserved by nuclear Ran GTPase-activating protein which catalyzes the production of RanGTP from RanGDP [4]. The maintenance of this RanGTP gradient is essential for driving the directionality of the XPO1 cycle [4]. Once XPO1 interacts with RanGTP, it forms a stable complex that allows for the docking of XPO1 cargo molecules onto the nuclear export signal binding cleft on XPO1. This ternary complex is then able to migrate through the NPC into the cytoplasm. Upon reaching the cytoplasm, RanGTP is hydrolyzed into RanGDP by the Ran guanine nucleotide exchange factor and the complex dissociates thereby depositing the XPO1 cargo molecule in the cytoplasm. Proteomics studies using a range of cell subtypes estimate the XPO1 exportome to range between 200 and 1000 unique cargo molecules [5]. Despite this seemingly large number of potential cargo molecules, to date, many of these are yet to be fully validated and large-scale proteomics studies to identify cargo molecules of critical importance to specific malignancies are lacking. This aside, a number of XPO1 cargo molecules have been validated in cancer cells lines and include some well-known nuclear tumour suppressors such as Rb, p27, p21 and the aforementioned p53. Given that the export of nuclear tumour suppressor proteins to the cytoplasm is a potential mechanism to compromise their function, the inhibition of XPO1’s nuclear export function is an attractive target for anticancer therapies that restore nuclear tumour suppressor activity by preventing nuclear export [6]. Subsequent drug development initiatives have given rise to a class of small molecules known as the selective inhibitor of nuclear export (SINE) compounds which includes the novel XPO1 inhibitor selinexor.

## 2. XPO1 Cargo in NHL

### 2.1. Proteins as XPO1 Cargo

XPO1 cargo has been examined broadly using yeast and HeLa cells [5], but little work has examined cargo in NHL. Immunofluorescence staining, Western blotting, and immunoprecipitation assays have identified XPO1 cargo in NHL by inhibiting nuclear export using the small molecule drug KPT-185 [7] and by monitoring subsequent changes in protein localization. This has led to the identification of tumour suppressor proteins including p53 and FOXO1 as XPO1 cargo in NHL, which may have implications for cancer growth and chemotherapy resistance. A full list of XPO1 cargo identified is lymphoma is available in Table 1.

The tumour suppressor proteins p53, p73, p21, p27, and FOXO1 inhibit cell cycle progression and are activated following DNA damage [11,12]. These proteins exert their functions in the nucleus of the cell, recognizing damaged DNA, stalling the cell cycle while the DNA is being repaired and initiating apoptosis if needed [12]. These proteins play an important role in responding to chemotherapy and radiation, which act by damaging DNA; as such, cancer cells have been found to hijack these processes by XPO1-mediated export of tumour suppressor proteins [7]. As seen in lymphoma, XPO1-mediated mislocalization prevents these proteins from exerting their anti-cancer functions, which potentially leads to disease progression and chemotherapy resistance [13,14]. This is further supported by studies finding that XPO1 inhibition using SINE compounds restores nuclear localization of tumour suppressor proteins and sensitizes the cancer cells to chemotherapy [15].

Other studies have inhibited XPO1 using the novel small molecule KPT-330 and used immunofluorescence staining or fluorescent microscopy to observe changes in protein localization [8,16]. These studies have identified XPO1 cargo relevant to the immune response and inflammation, including inhibitor of kappa B (IkB) [8] and STAT transcription factors [9]. 

The NFkB pathway is central to many aspects of cancer progression and negatively regulated by IkB [17]. While this processes is highly complex, the transcription factor NFkB promotes cancer development and growth by activating pro-inflammatory genes that lead to cell activation and growth, as well as signaling molecules that can promote cancer proliferation and invasion of normal tissues [18]. IkB inhibits NFkB’s transcriptional function by blocking NFkB’s nuclear localization sequence (NLS) and results in its retention in the cytoplasm, where it cannot activate inflammatory genes and pathways [18]. While IkB acts in the cytoplasm, normal cells have a balance of nuclear and cytoplasmic localization of this protein, and this homeostatic balance is critical for ensuring proper IkB expression and activity; too much cytoplasmic IkB results in its degradation via proteasomes, decreasing the availability and activity of IkB and increasing NFkB nuclear localization and activity [18]. Cancer cells therefore use XPO1 to excessively export IkB to the cytoplasm and disrupt homeostasis, increasing its degradation and resulting in excessive NFkB signaling. This process has been found to promote cancer progression in certain lymphomas. Selinexor-mediated inhibition of IkB export is capable of restoring this homeostatic balance, downregulating the NFkB pathway and promoting anti-cancer activity [17].

Signal transducers and activators of transcription (STATs) belong to a family of transcription factors with a variety of functions. While some STAT proteins have anti-cancer activity, others act to promote cancer growth and progression by regulating genes involved in cell growth, inhibition of apoptosis and immune evasion [19]. STAT3 and STAT6 have lymphoma-promoting properties due to their activation of genes encoding for proteins such as MYC, BCL-XL and CYCLIND1 [19]. STAT6 is also mutated in 11–36% of diffuse large B-cell lymphomas (DLBCL), follicular lymphomas (FL), and primary mediastinal B-cell lymphomas (PMBCL) [20,21,22]. These STAT mutations frequently occur in the DNA-binding domain of the protein and appear to increase the activation of oncogenic target genes [20]. STAT transcription factors function in the nucleus but are activated in the cytoplasm by a phosphorylation cascade mediated by cytokine receptors; lymphoma cells have been found to use XPO1 to increase nuclear export of STAT6 resulting in elevated levels of activated pSTAT proteins that then translocate to the nucleus to transcribe cancer-promoting genes [19]. STAT3 has been identified as XPO1 cargo in breast cancer [23], and although it has not yet been validated in lymphoma, its importance in immune cell maturation and activation and presence of a bona fide XPO1 binding NES suggests that it is most likely an XPO1 cargo molecule in NHL. 

### 2.2. RNA as XPO1 Cargo

More recently, RNA as XPO1 cargo has been gaining attention and interest. As nucleic acids to not contain canonical XPO1 NESs, RNA export is facilitated by associating with a wide range of adaptor proteins that contain nuclear export sequences (NESs) that allow them to be identified and exported by XPO1 [24]. Several studies have identified a subset of messenger RNA (mRNA), as well as pre-micro RNA (pre-miRNA), ribosomal RNA (rRNA), and small nuclear RNA (snRNA), that are exported in cancer, albeit not specifically in lymphoma [24].

While most mRNA is exported via the TAP/NXKF1 pathway, some specific types of mRNA are exported via XPO1 [25]. For example, AU-rich element (ARE)-containing mRNAs are exported with the help of the human antigen R (HuR) adaptor protein, and the tissue-specific factor NXF3 may serve as an adaptor for tissue-specific mRNA export [26]. However, most XPO1-exported mRNAs utilize the eukaryotic translation initiation factor eIF4E and the leucine-rich pentatricopeptide repeat protein (LRPPRC) adaptor [25,26]. LRPPRC recognizes and interacts with eIF4E in the nucleus, where it mRNA via the 5′ methyl-guanosine cap, binding prior to forming a complex with XPO1 [27]. Once in the cytoplasm, eIF4E initiates ribosomal translation of the mRNA [28]. Interestingly, most mRNA exported via this eIF4E-dependent XPO1 pathway are oncogenic, being involved in cell cycle progression, proliferation, and metastases [29]. Known transcripts exported and translated with the help of eIF4E include VEGF, which facilitates angiogenesis to increase tumour blood supply, BCL-2, which plays a role in cell survival, and growth stimulators c-Myc and Cyclin D1 [28]. Further evidence for these processes have been demonstrated in mantle cell lymphoma (MCL), where immunoblotting has found that oncogenic mRNA encoding cell proliferation factors such as Cyclin D1, PIM-1, and c-Myc are indeed exported via XPO1 [10]. Exporting these cancer-promoting transcripts allows for their increased expression, which increases the proliferative capacity and aggression of lymphoma by aiding in survival, cell division invasion, and metastases [24].

Furthermore, XPO1 has been found to facilitate the nuclear export of unprocessed viral mRNA [26]. Transcripts that have not been completely processed via splicing are able to be exported by XPO1, bypassing nuclear quality control mechanisms in order to provide the cell with abundant mRNA and proteins in times of stress [26]. While the relevance of this to lymphoma has not been directly investigated, this is important to consider given that many lymphomas such as Burkitt, T cell, and NK cell malignancies are associated with infection with Epstein–Barr Virus (EBV) [30]. The utilization of XPO1 to bypass these quality control and homeostatic mechanisms for mRNA export may therefore play a role in lymphomagenesis, and this phenomenon constitutes an interesting avenue that should be explored both in the context of and independently of EBV infection.

Several bodies of work in the field have identified other potential RNA cargo. While the additional RNA cargos were not identified as XPO1 targets in lymphoma, they bear mentioning here as potentially important cargo in NHL despite the lack of definitive, supporting evidence Ribosomal RNA (rRNA), small nuclear RNA (snRNA), and micro RNA (miRNA) are exported into the cytoplasm by XPO1 with the help of adaptor proteins such as NMD3, the cap binding protein CBC, and the phosphorylated adaptor for RNA export (PHAX) [31]. Gene silencing pre-miRNAs have been identified as cargo in kidney and colon cancer cells [32], so there is reason to hypothesize that they may also be XPO1 targets in NHL. Future research should investigate the role of XPO1-mediated miRNA export in lymphoma, in conjunction with the identification of miRNA targets explore the potential role of an XPO1-miRNA axis NHL growth and treatment resistance.

Similarly, rRNA and snRNA has been identified as cargo in yeast [33], but research implicating delineating these processes in NHL is lacking. Since rRNA is a component of ribosomal subunits, which are also exported by XPO1 [34], lymphoma cells may be exporting abundant amounts of these translation machineries to aid in the increased expression of oncogenic proteins. Similar to mRNA and eIF4E, increased export of translation machinery may provide cells with an advantage in times of stress by ensuring proteins are being created for cell growth and proliferation, but this is something that needs to be further investigated to determine its implications for lymphoma.

## 3. Preclinical Data on Selinexor as a Monotherapy

Our current understanding regarding selinexor’s mechanism of cellular cytotoxicity in a preclinical context is based on a collection of studies interrogating the activities of the various SINE compounds. KPT-185 is commonly used for in vitro experiments due to its high potency, however, its murine and human in vivo use is limited by its poor pharmacokinetics and oral bioavailability [6]. For these experiments, KPT-251 and KPT-276 are more commonly used. The use of selinexor is most often seen in clinical trials that have been guided by the results of preclinical studies using either selinexor or other SINE compounds. A comprehensive list of the preclinical studies investigating selinexor and related SINE compounds as a monotherapy in NHL is available in Table 2.

### 3.1. Preclinical Trials Investigating SINE Compounds in NHL

In vitro experiments with KPT-185 have demonstrated its cytotoxicity and potential mechanisms for its anti-cancer activity in NHL. Various NHL cell lines and patient tumour samples treated with KPT-185 demonstrated growth inhibition, reduced cell viability, and induced apoptosis after 72 h [35]. Analysis via Western Blot found that KPT-185 induced cleavage of caspase 3, caspase 8, caspase 9, and PARP in a dose and time dependent manner, indicating that the drug may initiate apoptosis using several mechanisms in parallel. The drug also arrested cells in the G1 phase of the cell cycle, as seen by flow cytometry [35].

Interestingly, Western Blot and PCR analyses in these experiments also found that KPT-185 decreased XPO1 protein expression, but increased XPO1 mRNA expression [35]. It was hypothesized that the decreased protein expression was due to proteasome-mediated degradation. Indeed, when KPT-185 was combined with proteasome inhibitor bortezomib, this reduction in protein expression was not observed. 

Xu et al. examined KPT-185 efficacy in the context of wild type and mutant p53. Mutations in this tumour suppressor protein are seen in about 30% of NHL cases, and are associated with poor prognosis due to treatment resistance [37]. Preclinical studies using NHL cell lines with differing p53 functions found that KPT-185 induced apoptosis and inhibited growth of NHL cells, but not normal cells [7]. These results appeared to be independent of p53 function and mutational status, although other studies using MCL cell lines have found that while the drug is still effective in p53-mutant cells, it is not as potent as in cells harbouring wild-type p53 [13,38].

Furthermore, Western Blot and immunofluorescence staining determined that increasing KPT-185 concentrations resulted in an increase in nuclear localization of major tumour suppressor proteins such as p21, p27, p73, and FOXO3, indicating that a restoration of the nuclear pool of these proteins may play an important role in cytotoxicity, and may be of particular importance in the context of cells with mutant p53 [7]. Indeed, siRNA knockdown of each of these other tumour suppressor proteins reduced the induction of apoptosis in these models, providing further evidence for this interpretation.

The significance of the tumour suppressor protein family in promoting cancer cell death was further observed in MCL, as KPT-185-mediated nuclear restoration of p53 activated transcription of its target genes such as FAS, PUMA, and DR5, which act to promote apoptosis [13]. Additionally, and similar to previous studies, KPT-185 treatment also arrested NHL cells in the G0-G1 phase of the cell cycle, halting their growth and initiating apoptosis [7]. This effect was not seen when tumour suppressors p53 or p73 were knocked-down using siRNA. Together, these experiments provide evidence that the broad effects of KPT-185 on tumour suppressors as a whole plays an important role in its anti-cancer activity, especially in the context of mutated p53.

In vivo, mouse models treated with KPT-276 demonstrated shrinkage of their NHL tumours without any visible signs of toxicity or excessive weight loss in these animals [7,35]. The oral treatment showed equivalent anti-cancer activity when compared to the standard chemotherapy regimen used for NHL. Upon histological examination of the tumours derived from mutant p53 cell lines, researchers found that there was a universal increase in the expression of the tumour suppressor p73 [7]. Furthermore, this elevated p73 expression was coupled with a reduced index of proliferation, providing further evidence that nuclear restoration of tumour suppressors is a key player in the efficacy of KPT-276.

### 3.2. Preclinical Studies Investigating Selinexor

While most preclinical studies in lymphoma utilize KPT-185 in vitro and KPT-276 in vivo, studies using selinexor (KPT-330) provide the greatest insight on the efficacy of SINE molecules prior to initiating clinical trials as they are directly translatable to the compound that has been used in humans. To date, the preclinical studies for selinexor in lymphoma have mainly examined cytotoxicity and cell cycle arrest in various subtypes of NHL [36]. Here, selinexor demonstrated strong anti-proliferative and cytotoxic effects in mantle cell lymphoma (MCL) and T-cell lymphoma (TCL), but significantly higher doses were required to achieve anti-proliferative effects in DLBCL. Similar results were observed when examining selinexor’s impact on cell cycle progression, as it induced G1 cell cycle arrest in MCL and TCL, but not DLBCL. Taken together, these results suggest that although selinexor has been approved to treat DLBCL [39], its use in other subtypes of NHL such as MCL should be further investigated due to its potential to have a greater impact on arresting the cell cycle and inducing apoptosis.

## 4. Preclinical Data on Selinexor in Combination with Other Agents

A list of combination therapies with selinexor for NHL can be found in Table 3. At the time of writing, the preclinical data combining selinexor with chemotherapy or targeted agents has yielded encouraging results.

### 4.1. Selinexor + BTK Inhibitors

Preclinical studies have examined selinexor in combination with the Bruton’s Tyrosine Kinase (BTK) inhibitor zanubrutinib in DLBCL and mantle cell lymphoma (MCL) [40]. BTK is a member of the tyrosine protein kinase family that is activated by the B-cell receptor (BCR) signaling cascade [44]. Once activated, BTK is a key player in the activation of various oncogenic pathways such as the pro-inflammatory NFκB pathway and the cell-cycle progressing mitogen-activated protein kinase (MAPK) pathway [44]. BTK also amplifies signals from the BCR that are involved in B-cell survival, migration, and proliferation, positioning it as a an important oncoprotein in B-cell lymphomas.

In this light, zanubrutinib has found application as a compound used to treat B-cell lymphomas characterized by chronic BCR signaling and several studies using zanabrutinib in relapsed/refractory MCL can be found in the literature [44]. Tarantelli et al. tested the combination of zanubrutinib and selinexor in MCL cell lines to determine if selinexor can increase the efficacy of BTK inhibition by restoring nuclear localization of oncogenic proteins downstream of BCR signaling [40]. Since the aggressive Activated B Cell (ABC) subtype of DLBCL also demonstrates constitutive activation of BCR signaling [45], the combination was also tested on ABC-DLBCL cell lines.

In vitro studies using three ABC-DLBCL and two MCL cell lines involved treatment with increasing concentrations of selinexor and zanubrutinib for 72 h and measuring its effects on cell growth. The combination was found to be synergistic at lowering cell count in both MCL lines and 1 DLBCL line, and additive in another DLBCL line. The one DLBCL cell line where the combination had no significant benefit was the zanubrutinib-resistant SUDHL-2 line, indicating that while selinexor is able to be effectively combined with zanubrutinib, it is not able to overcome resistance to the drug [40].

To determine the mechanism by which this combination was effective, Tarantelli and colleagues examined the drugs’ effects on the cell cycle. Whereas individual treatments with selinexor or zanubrutinib mostly arrested cells in the G1 phase of the cell-cycle, the combination resulted in most cells entering the sub-G1 phase, where they subsequently underwent apoptosis [40]. While it is unclear what proteins selinexor localized to the nucleus to achieve this effect, these results suggest that XPO1 cargo retention in the nucleus is contributes to the cytotoxicity and apoptosis in the context simultaneous BTK inhibition.

### 4.2. Selinexor + Proteasome Inhibitors

Preclinical studies have investigated selinexor in combination with the proteasome inhibitor bortezomib, which antagonizes the catalytic activity of the 26S proteasome and prevents activation of pro-inflammatory transcription factor NFκB [46]. This drug is most commonly used to treat multiple myeloma, however, increasing evidence is supporting its potential use in treating ABC-DLBCL with constitutively activated NFκB [47]. In this case, bortezomib is able to inhibit the NFκB pathway by preventing the degradation of its inhibitor IκB-α [18]. This results in the cytoplasmic retention of NFκB thus inhibiting its function as transcription factor, inhibition of B cell growth, and induction of apoptosis [47].

IκB-α is also a known cargo molecule of XPO1 in hematological malignancies [17]. Even though it acts in the cytoplasm to block the nuclear localization sequence (NLS) of NFκB, healthy cells have a balance of IκB-α in both the nucleus and cytoplasm to signal that the cell is at a homeostatic equilibrium and to prevent excess inactivation of IκB-α via cytoplasmic proteasomes [18]. By continuously exporting IκB-α to the cytoplasm, cancer cells disrupt this balance within the cell, increasing degradation and deactivation of this inhibitor and therefore increasing activity of the NFκB pathway. By inhibiting XPO1, selinexor has been demonstrated to restore nuclear localization of IκB-α, downregulating the NFκB pathway and promoting apoptosis in hematological malignancies [17].

Due to their anti-cancer effects via IκB-α activation and NFκB downregulation, Kashyap et al. investigated the efficacy of combining bortezomib and selinexor in vitro and in vivo [46]. In vitro studies using DLBCL cell lines demonstrated that the combination was synergistic at lowering cancer cell viability after 72 h of treatment. As expected, the combination enhanced nuclear retention of IκB-α, making this the most likely mechanism of anti-cancer activity. Interestingly, the combination also decreased transcription of NFκB, providing further evidence for the strength of this combination, although the reason for this effect is not entirely clear.

Interestingly, contrasting results were observed by Abeykoon et al. [36], who found that selinexor in combination with bortezomib did not produce synergistic effects in DLBCL, but rather in MCL and TCL, for reasons that have yet to be explained. This cell line model suggested that selinexor’s synergy with proteasome inhibitors may be stronger in other subtypes of DLBCL and should therefore be further explored. 

### 4.3. Selinexor + Salicylates

Salicylates are a class of anti-inflammatory drugs that have been found to relocalize p65, one of the subunits of NFκB, to the nucleolus in cancer cells [48]. Although these drugs have not been examined in-depth as cancer treatments, their ability to modulate nuclear localization prompted researchers to investigate the effects of combining XPO1 inhibitors with choline salicylate (CH), a drug commonly used to treat rheumatoid arthritis [41]. As expected, CH alone had no cytotoxic effects on MCL cell lines but demonstrated synergistic effects when combined with selinexor in MCL or DLBCL cell lines. In vivo, the combination of selinexor and CH significantly decreased tumour growth and volume compared to either treatment alone and no significant adverse events were observed in the mouse models. 

The interactions between these two drugs and the mechanisms behind their synergy may be explained by their mutual actions in restoring nuclear localization of various cargo molecules. This is further supported by the investigators reporting that the combination therapy resulted in complete nuclear localization of an engineered reporter construct encoding green fluorescent protein (GFP) with a NLS, whereas selinexor alone resulted in incomplete nuclear localization. CH also acts as a modulator of nuclear export in, but the mechanisms underlying this process are poorly understood. However, its potential to restore nuclear localization of various proteins makes it an interesting candidate to potentiate selinexor’s mechanism of action. 

The combination also enhanced the degradation of XPO1 in a proteasome-dependent mechanism, as reduced protein levels were detected via Western blotting, and concurrent treatment with bortezomib prevented this reduction [41]. Additionally, proteins involved in oncogenic pathways such as CYCLINB1 also had reduced expression when the drugs were combined in comparison to either treatment alone. As such, the combination induced cell cycle arrest in the S phase. The investigators noted that the combination also resulted in decreased expression of Rad51, a protein involved in repairing damaged DNA, and therefore resulted in deficiencies in homologous recombination-mediated repair leading to apoptosis. Rad51 has not been identified as an XPO1 cargo molecule, but prior work in acute myeloid leukemia has demonstrated that XPO1 inhibition via selinexor is able to decrease the transcription of Rad51 mRNA transcription resulting in lower levels of protein expression, thus presenting a potential explanation for this observation [49]. 

### 4.4. Selinexor + BCL-2 Inhibitors

Venetoclax is a B cell lymphoma-2 (BCL-2) inhibitor used to treat various types of leukemia and is but actively being investigated as a potential treatment for DLBCL due to the role of BCL-2 in this malignancy. The BCL-2 protein is involved in inhibiting apoptosis and is normally expressed during B-cell maturation [50]. Due to its anti-apoptotic effects, BCL-2 is commonly overexpressed in hematological malignancies, including DLBCL [50]. Furthermore, alterations in BCL-2 expression can be driven both by genomic abnormalities and transcriptional activation. Twenty percent of DLBCL cases are characterized by a BCL-2 translocation, where the gene is fused to immunoglobulin healthy chain gene enhancers, resulting in increased transcription at this active promoter region [50]. In other cases, chromosomes carrying duplications of the BCL-2 gene lead to its amplification and increased expression [50]. Transcription of the of the BCL-2 gene is also regulated by NFκB which is often dysregulated and aberrantly overexpressed in many B-cell malignancies consequently resulting in BCL-2 overexpression [50].

Recognizing that the NFκB pathway is involved in both the nuclear export and activation of BCL-2, Elloul et al. conducted in vitro and in vivo studies examining the effect of combining selinexor with venetoclax [42]. The combination of these two molecules had synergistic effects in reducing DLBCL cell viability and inhibiting their growth. In addition, combining selinexor and venetoclax in NHL xenograft mouse models resulted in a further 30% shrinkage in tumour volume as compared to venetoclax monotherapy. Immunoblot and RT-PCR experiments also found that the combination treatment reduced BCL-2 protein and mRNA expression, respectively. A potential explanation for these findings may lie in selinexor’s ability to inhibit NFκB-mediated transcription of BCL-2 thus allowing for greater venetoclax occupancy of BCL-2 and thereby potentiating its inhibition. Conversely, as both NFκB and BCL-2 drive critical survival mechanisms in NHL, simultaneously targeting both pathways may be beneficial independently of the relationship between NFκB and BCL-2. Irrespective of the underlying mechanism, these results provide strong evidence that venetoclax and selinexor form a highly active combination in these NHL models. As NHLs with dysregulated BCL-2 for a subset of patients who typically respond poorly to standard of care therapy, venetoclax-selinexor combinations may be beneficial in this patient population providing the expected toxicities can be managed accordingly. 

### 4.5. Selinexor + Chemotherapy

Chemotherapy treatments such as bendamustine work by damaging the cells’ DNA. Bendamustine specifically alkylates bases and creates cross-links between DNA strands to cause breaks that the cancer cells may not be able to repair and may drive them to undergo apoptosis [51,52]. Bendamustine induced DNA damage is detected by nuclear tumour suppressor proteins, such as p53, which prevents the cell from dividing until the damaged DNA is repaired or the cell undergoes apoptosis. However, DLBCL and other types of cancer are occasionally refractory or can acquire resistance to chemotherapy by hijacking the DNA damage repair pathway both through p53 dependent and independent mechanisms. NHL cells have been shown to downregulate p53 expression, acquire loss-of-function/change of function mutations to p53, or export p53 to the cytoplasm via XPO1 thereby inhibiting its function in the DNA damage response pathway, preventing cell cycle arrest and apoptosis in response to DNA damage [14].

Preclinical studies have determined that nuclear localization of p53 can be restored with selinexor [14], prompting Elloul et al. to investigate if combining bendamustine with selinexor would potentiate the effects of selinexor-mediated inhibition of XPO1 [42]. Although it is not FDA approved for DLBCL, bendamustine is commonly used off-label in the 2nd line setting for DLBCL patients who are ineligible for a stem cell transplant, with a response rate of 55% [53,54]. It was therefore of interest to investigate whether selinexor could enhance the efficacy of bendamustine via nuclear retention of p53. Indeed, the combination had synergistic effects on reducing DLBCL cell viability and inhibiting their growth, and also resulted in an additional 30% shrinkage in tumour volume in mouse models compared to either treatment alone [42]. Although the authors did not report the proportion of mice that responded to the combination treatment, the synergistic effects on tumour shrinkage indicate a potential benefit of combining bendamustine with selinexor, likely due to the nuclear retention of p53 sensitizing the cells to chemotherapy.

Gemcitabine is a treatment commonly used as a salvage therapy for relapsed NHL [36]. This chemotherapy acts by terminating DNA synthesis during the replication process, creating an incomplete DNA strand that activates p53-dependent cell cycle arrest and apoptosis processes [55]. When combined with selinexor in various NHL cell lines, the combination exhibited moderate-to-strong synergistic effects, specifically in MCL and TCL, likely due to activation of p53 [36]. Interestingly, synergy was not observed in DLBCL, in contrast to previous findings examining chemotherapy combinations. Although the authors did not elaborate on the possible reasons behind these findings, the treatment’s effects in MCL and TCL warrant further exploration. 

Finally, positive preclinical studies investigating selinexor combined with an immuno-chemotherapy combination used as part of the current standard of care for NHL (rituximab, cyclophosphamide, doxorubicin, vincristine, and prednisone; commonly referred to as R-CHOP) have inspired early phase clinical trials for this combination [43]. Frontline R-CHOP therapy can cure approximately 50–70% of patients, however, an estimated 30–50% either relapse or are refractory to primary treatment [43,56]. The reasons for relapse and treatment resistance are thought to be quite complex and have not been fully elucidated. A variety of factors are likely to play a role in treatment failure including, but not limited to, immune evasion, tumour genetics, dysregulated cell signaling pathways, biology attributed to an altered tumour microenvironment and dysfunctional DNA repair pathways. Given the breadth of XPO1 cargo molecules and the potentially pleotropic effects of selinexor on cellular function in NHL, Seymour et al. examined whether inhibition of nuclear export via selinexor could restore the balance of these pathways and improve the response rates in the context of a selinexor R-CHOP combination [43].

In vitro, the combination of cyclophosphamide, doxorubicin, and vincristine (CHO) enhanced apoptotic effects and inhibited cell proliferation and resulted in increased in PARP cleavage to initiate cell death [43]. When examining the potential mechanism by which this effect occurred, researchers found that selinexor increased expression of phosphorylated ERK (pERK). This may seem paradoxical since MAPK is involved in cell cycle progression; however, it also induces expression of CD20, the target of rituximab [43]. It is estimated that 30% of relapsed/refractory cases of NHL demonstrate a loss of CD20 expression, a mechanism used to evade the anti-cancer effects of rituximab; therefore, increasing CD20 expression via pERK can help restore sensitivity to the treatment [43]. Indeed, researchers found that selinexor not only increased levels of pERK, but also increased levels of CD20 expression, suggesting a possible mechanism for the enhanced effects of the combination therapy. However, it remains unclear how selinexor was able to increase pERK expression to achieve these results. further investigation is required to elucidate the mechanism by which selinexor modulates ERK activation. 

The combination of selinexor and CHO was also effective in vivo as demonstrated by xenograft mouse models [43]. SCID mice injected with WSU-FSCCL follicular lymphoma cells were given either no treatment, selinexor, CHO alone, or a combination of selinexor and CHO demonstrated a 64% longer median overall survival compared to those given CHO alone. Furthermore, 3 out of the 7 mice given the combination had undetectable disease 160 days post-tumour inoculation and were deemed to be in long-term remission. This is compared to only 1 out of 7 mice given selinexor alone, and 2 out of 7 mice given CHO alone, obtaining these responses, a difference reported as statistically significant. 

## 5. Clinical Trials with Selinexor

The positive results from preclinical studies have prompted the investigation of selinexor in small phase I/II clinical trials for various types of NHL. A full list of completed clinical trials is available in Table 4, and ongoing trials can be viewed in Table 5.

### 5.1. Selinexor as a Monotherapy

The first in-human phase I study investigating selinexor as a monotherapy enrolled 79 patients with relapsed/refractory (R/R) NHL [57,61]. DLBCL was the most common lymphoma subtype in this cohort, but patients with FL, chronic lymphocytic leukemia (CLL), MCL, TCL, Burkitt lymphoma (BL), and marginal zone lymphoma (MZL) were also included. This dose-escalation study examined safety and efficacy of selinexor, as well as the pharmacokinetics (PK) and pharmacodynamics (PD). The objective response rate (ORR) was 31%, with 6% of patients demonstrating a complete response (CR), and 26% indicating a partial response (PR).

Several toxicities were noted in this trial that necessitated dose reductions. These toxicities were mainly hematologic in nature, including thrombocytopenia, neutropenia, and anemia. However, there was only 1 dose-limiting toxicity (DLT) of grade 4 thrombocytopenia, and the maximum tolerated dose (MTD) was not reached. Due to doses above 40 mg/kg^2^ resulting in significantly increased incidences of adverse events and reduced time on therapy, the recommended phase II dose (RP2D) identified was 35 mg/kg^2^, simplified to a flat dose of 60 mg [57]

Similar to the preclinical studies, PD analyses on the tumour biopsies found that selinexor decreased protein expression as measured by immunofluorescent staining of BCL-2, BCL-6, and c-Myc, and restored nuclear localization of XPO1 cargo such as p53 [57]. The results of the correlative analysis using paired pre-treatment and on-treatment tumour biopsies are consistent with results from preclinical studies thus providing in vivo evidence corroborating selinexor’s mechanism of action in NHL.

The subsequent phase II trial SADAL evaluated weekly 60 mg and 100 mg doses of selinexor in patients with R/R DLBCL [58]. Similar to the phase I trial, the primary outcome of interest was ORR, and other outcomes of interest included duration of response, disease control rate, and PK/PD analysis. The ORR was 28%, with a CR of 12% and PR of 17%. Histological and PD analyses found that this rate did not differ significantly based on the cell of origin, consistent with the results found in preclinical studies [62]. It is also worth noting that patients with high levels of c-Myc had a mere 13% ORR, whereas patients with low levels had a significantly higher ORR of 42% [58].

AEs were seen in 98% of patients but were reversible with supportive care or dose modification [58]. However, due to the higher rate of AEs in the 100 mg cohort with no significant added response benefit, the 100 mg group was discontinued after an interim analysis. The safety and efficacy of the treatment in the 60 mg group resulted in selinexor’s approval by the FDA for adult patients with R/R DLBCL after at least 2 prior lines of therapy [39].

### 5.2. Selinexor in Combination with Other Anti-Cancer Agents

The promising results from the preliminary clinical trials using selinexor monotherapy in NHL supported subsequent investigations for use of selinexor in combination with other anti-cancer agents. Based on the positive results of preclinical studies examining the combination of selinexor and BTK inhibitors, ref. [40] a recent phase I clinical trial examined the combination of selinexor and daily BTK inhibitor ibrutinib in 33 patients with R/R NHL and chronic lymphocytic leukemia (CLL) [63]. Using this combination therapy, 81% of patients achieved disease control, with 48% reaching stable disease and 33% achieving either a CR or PR, even in the case of BTK mutations that result in ibrutinib resistance when administered as a single agent. This combination was deemed to be safe and effective, with only 2 patients experiencing a dose-limiting toxicity (DLT).

The recent Phase I clinical trial testing selinexor and R-CHOP in NHL involved patients receiving six 21-day cycles of R-CHOP and either 60 mg or 80 mg of weekly selinexor, followed by weekly selinexor maintenance for 1 year [43]. 12 patients were enrolled in the study; 10 had DLBCL and 2 had follicular lymphoma (FL). The combination was found to be safe and effective, with the majority of adverse events being grade 1 or 2 and overall response rate (ORR of 100%, with 9 out of 10 evaluable patients achieving a complete response. The authors suggested a recommended phase II dose (RP2D) of 60 mg of selinexor instead of 80 mg, since the 80 mg cohort had a higher frequency of dose reductions, discontinuations, and Grade 3 Adverse Events (AEs) with no significant added benefit compared to the 60 mg group.

Patients also had blood samples taken throughout the course of their treatment for PD and biomarker analysis to determine the combination’s mechanism of efficacy. In keeping with previous preclinical studies [43], this combination resulted in elevated levels of pERK and increased both CD20 mRNA and protein expression. The combination also resulted in a downregulation of oncogenic BCL-2 and AKT, as well as an upregulation of the pro-apoptotic protein BAX. It remains unclear as to how selinexor modulates the observed changes in protein and transcript expression in these NHL subtypes. A fuller characterization of XPO1’s cargo molecules (both proteins and nucleic acids) in NHL would be useful to elucidate the mechanisms that drive these phenomena. This knowledge gap aside, it is clear that the combination of selinexor with R-CHOP constitutes an active regimen with manageable toxicities. Subsequently, a multi-centre, frontline Phase II study evaluating this combination in NHL has been initiated and is currently accruing patients (NCT03147885).

Finally, a phase I study investigated the combination of selinexor with dexamethasone, ifosfamide, carboplatin and etoposide (DICE) in 11 patients with R/R peripheral T cell lymphoma (PTCL) or natural killer/T cell lymphoma (NKTL) [60]. The combination was effective, with an ORR and CR rate of 91% and 82%, respectively. However, the combination was poorly tolerated, with 45% of patients discontinuing treatment for reasons other than disease progression or a lack of response. Given the toxicities associated with this combination, additional studies will be needed if selinexor is to be incorporated into similar chemotherapy regiments to treat these lymphomas.

## 6. Conclusions and Future Directions

XPO1 functions as an exporter of a wide range of cargo molecules that aid in the growth and progression of cancer. SINE compounds, such as selinexor, that are potent XPO1 inhibitors are able to simultaneously target numerous pathways in NHL cells, leading to cytotoxic effects and sensitization to other anti-cancer agents. The pleotropic properties of XPO1 inhibitors results in the modulation of multiple downstream pathways by restoring imbalances in cargo protein and RNA localization. These properties make them interesting candidates for combination therapies. Defining appropriate and rational combination therapies using selinexor or other SINE compounds in NHLs is dependent on a multitude of factors and will need to be determined empirically.

NHL is a highly heterogeneous disease, with many patients presenting with a spectrum of genetic abnormalities and protein expression profiles that affects response to treatment and prognosis. For example, each case of the DLBCL subtype of NHL presents with an estimated 75 different mutations, indicating a prominent role for genetic and biomarker testing to inform individual treatments for this disease [56]. It is evident that there is no one-size-fits-all approach to effectively treating NHL, especially in relapsed/refractory settings. Novel therapies and drug combinations are needed for patients with aggressive disease and, if possible, should be tailored to the unique features of their particular NHL subtype. In the context of selinexor and the family of SINE molecules in general, further research is needed to better understand XPO1 biology in lymphoma and how this is modulated by selinexor’s ability to inhibit XPO1 function. One approach to achieving this would be to better identify and validate critical XPO1 cargo molecules in different subsets of NHL. This may uncover avenues and rationales for testing novel combinations therapies. Another method involves examining the effect of combinations on nuclear localization of proteins relevant to NHL, and testing combinations on different types of high-grade lymphomas such as those with BCL-2 and c-Myc rearrangements to determine their efficacy in the context of features that drive aggressive disease subtypes. Lastly, given the expanding role of immunotherapies in NHLs, understanding how selinexor directly affects the function of immune effectors and how NHL ells present themselves to the immune system will be critical to effectively sequence or combine SINE molecules with bispecific antibodies, engineered cell therapies, checkpoint inhibitors and novel any novel immunotherapies that show promise for the treatment of NHL. 

## Figures and Tables

**Table 1:** XPO1 Cargo Identified in NHL.

Cargo Protein	Cargo Function	Discovered in	Methods
p53 [7]	Tumour suppressor protein	Non-Hodgkin lymphoma	Immunofluroescence staining, Western blotting, and immunoprecipitation after NHL cells were treated with nuclear export inhibitor KPT-185
p73 [7]	Tumour suppressor protein	Non-Hodgkin lymphoma	Immunofluroescence staining, Western blotting, and immunoprecipitation after NHL cells were treated with nuclear export inhibitor KPT-185
p21 [7]	Tumour suppressor protein; CDK inhibitor	Non-Hodgkin lymphoma	Western blotting after NHL cells were treated with nuclear export inhibitor KPT-185
p27 [7]	Tumour suppressor protein; CDK inhibitor	Non-Hodgkin lymphoma	Western blotting after NHL cells were treated with nuclear export inhibitor KPT-185
FOXO3 [7]	Tumour suppressor protein	Non-Hodgkin lymphoma	Western blotting after NHL cells were treated with nuclear export inhibitor KPT-185
IkB [8]	Inhibitor of inflammation	Non-Hodgkin lymphoma	Fluorescence microscopy and flow cytometry in NHL cells treated with nuclear export inhibitor KPT-330
STAT6 [9]	Transcription factor for maturation of immune cells including B cells, T cells, and macrophages	Primary mediastinal B-cell lymphoma	Immunofluroescence staining with overlap showing interaction of XPO1 and STAT, and how nuclear export inhibitor KPT-330 disrupts this
Cyclin D1 mRNA [10]	Cell cycle progression factor	Mantle cell lymphoma	Immunoblotting after NHL cells were treated with nuclear export inhibitor KPT-185
PIM1 mRNA [10]	Cell cycle progression factor	Mantle cell lymphoma	Immunoblotting after NHL cells were treated with nuclear export inhibitor KPT-185
c-Myc mRNA [10]	Transcription factor for cell proliferation	Mantle cell lymphoma	Immunoblotting after NHL cells were treated with nuclear export inhibitor KPT-185

**Table 2:** Preclinical Data Investigating Selinexor and its Related Compounds as Monotherapies for Non-Hodgkin Lymphoma.

Preclinical Model	Lymphoma Type	Results	Significance
Cell lines and patient tumour samples [35]	Non-Hodgkin lymphoma	Induction of caspase and PARP cleavage initiating apoptosis	SINE-induced apoptosis is initiated by several mechanisms
Cell lines [7]	Non-Hodgkin lymphoma	Initiated apoptosis regardless of p53 function, but silencing other family members reduced efficacy	p53 and its family members play an important role in SINE-mediated cytotoxicity
Cell lines [13]	Mantle cell lymphoma	Initiated expression of anti-cancer proteins and induced apoptosis regardless of p53 function, but p53-mutant cells were less sensitive	p53 plays an important role in SINE-mediated cytotoxicity
Mouse models [7]	Non-Hodgkin lymphoma	Inhibition of tumour growth with anti-cancer activity equivalent to the chemotherapy standard of care (SOC), possibly due to enhancement of p73	Enhanced expression and activity of p73 as a result of SINE compounds may be a mechanism behind their strong anti-cancer activity; provides a direct comparison of the treatment’s efficacy to the SOC with positive results
Mouse models [35]	Non-Hodgkin lymphoma	Inhibition of tumour growth with minimal toxicity and weight loss	Demonstrates safety and efficacy in vivo
Cell Lines [36]	Non-Hodgkin lymphoma	Strong anti-proliferative effects and cell cycle arrest were observed in T-cell lymphoma and mantle cell lymphoma, with less pronounced effects in diffuse large B-cell lymphoma	Demonstrates selinexor’s anti-cancer effects at the molecular level and how they vary in different non-Hodgkin lymphomas

**Table 3:** Combination Therapies Tested Preclinically for Selinexor (S) in Non-Hodgkin Lymphoma.

Combination Therapy	Drug Class	Used in	Preclinical Model	Outcomes/Relevant Data
S + zanubrutinib [40]	BTK inhibitor	Diffuse large B-cell lymphoma	Cell lines	Synergistic effects on lowering cell count
S + zanubrutinib [40]	BTK inhibitor	Mantle cell lymphoma	Cell lines	Synergistic effects on lowering cell count
S + bortezomib [36]	Proteasome inhibitor	Non-Hodgkin lymphoma	Cell Lines	Synergistic cytotoxic effects were seen in mantle cell lymphoma and T-cell lymphoma, but not diffuse large B-cell lymphoma
S + gemcitabine [36]	Chemotherapy	Non-Hodgkin lymphoma	Cell Lines	Synergistic cytotoxic effects were seen in mantle cell lymphoma and T-cell lymphoma, but not diffuse large B-cell lymphoma
S + choline salicylate [41]	Anti-inflammatory	Non-Hodgkin lymphoma	Cell lines and mouse models	Synergistic effects on apoptosis and tumour shrinkage
S + venetoclax [42]	BCL-2 inhibitor	Diffuse large B-cell lymphoma	Cell lines and mouse models	Synergistic effects on apoptosis and tumour shrinkage
S + bendamustine [42]	Chemotherapy	Diffuse large B-cell lymphoma	Cell lines and mouse models	Synergistic effects on apoptosis and tumour shrinkage
S + CHO [43]	Chemotherapy combination	Non-Hodgkin lymphoma	Cell lines and mouse models	Synergistic cytotoxic effects, increased survival

**Table 4:** Completed Clinical Trials for Selinexor Treatment in Non-Hodgkin Lymphoma.

NCT	Treatment	Phase	Disease Type	Major Outcomes	Notable Toxicities
NCT01607892 [57]	Selinexor	I	Relapsed/refractory Non-Hodgkin lymphoma	ORR: 31% RP2D: 60 mg	1 DLT of grade 4 thrombocytopenia MTD: not reached
NCT02227251 [58]	Selinexor	II	Relapsed/refractory diffuse large B-cell lymphoma	ORR: 28%	Discontinuation of the 100 mg cohort due to higher toxicity with no significant added benefit
NCT02303392 [59]	Selinexor + ibrutinib	I	Relapsed/refractory Non-Hodgkin lymphoma/Chronic lymphocytic leukemia	DCR: 81% ORR: 33% SD: 48%	MTD: 40 mg selinexor + 420 mg daily ibrutinib DLT: experienced in 2/33 patients
NCT03147885 [43]	Selinexor + R-CHOP	I	Non-Hodgkin lymphoma	ORR: 100% CR: 90% RP2D: 60 mg selinexor	MTD: not reached Higher rate of grade 3 AEs in 80 mg group with no significant added benefit
NCT03212937 [60]	Selinexor + DICE	I	Relapsed/refractory peripheral T-cell lymphoma/natural killer T-cell lymphoma	ORR: 91% CR: 82%	MTD: 40 mg selinexor DLT: 2 patients 45% of patients discontinuing treatment for reasons other than disease progression/poor response

**Table 5:** Ongoing Clinical Trials for Selinexor Treatment in Non-Hodgkin Lymphoma.

NCT Identifier	Treatment	Phase	Disease Type
NCT02741388	Selinexor + R-DHAOx/R-GDP	II	Relapsed/refractory B-cell lymphoma
NCT02227251	Selinexor	IIb	Relapsed/refractory diffuse large B-cell lymphoma
NCT02471911	Selinexor + R-ICE	I	Relapsed/refractory B-cell lymphoma
NCT03212937	Selinexor + ICE	I	Peripheral T-cell lymphoma
NCT04442022	R-GDP +/− selinexor	II/III	Transplant/CAR-T ineligible relapsed/refractory B-cell lymphoma
NCT02303392	Selinexor + ibrutinib	I	Relapsed/refractory Non-Hodgkin lymphoma/Chronic lymphocytic leukemia
NCT02436707	Selinexor + R-GDP	II	Relapsed/refractory aggressive transplant ineligible B-cell lymphoma
NCT03992339	Selinexor	II	Relapsed/refractory diffuse large B-cell lymphoma
NCT03147885	Selinexor + RCHOP	Ib/II	B cell lymphoma
NCT04640779	Selinexor + choline salicylate	Ib	Relapsed/refractory Non-Hodgkin lymphoma
NCT03955783	Selinexor + venetoclax	Ib	High risk hematologic malignancies
